# Gene signature from cutaneous autoimmune diseases provides potential immunotherapy-relevant biomarkers in melanoma

**DOI:** 10.1038/s41598-023-42238-3

**Published:** 2023-09-12

**Authors:** Kyu-Hye Chun, Ye-Chan Park, Nahee Hwang, Bo Kyung Yoon, Jae-woo Kim, Sungsoon Fang

**Affiliations:** 1 Graduate School of Medical Science, Brain Korea 21 Project, Yonsei University College of Medicine, Seoul, 03722 Korea; 2 Department of Biochemistry and Molecular Biology, Yonsei University College of Medicine, Seoul, 03722 Korea; 3 Chronic Intractable Disease for Systems Medicine Research Center, Yonsei University College of Medicine, Seoul, 03722 Korea; 4 Severance Biomedical Science Institute, Gangnam Severance Hospital, Yonsei University College of Medicine, Seoul, 03722 Korea

**Keywords:** Cancer, Computational biology and bioinformatics, Immunology

## Abstract

Immune checkpoint inhibitors (ICIs) are promising agents for treating melanoma. Given that autoimmune skin diseases exhibit hyper immune reaction, investigation of immune cells from autoimmune skin disease is crucial to validate the effectiveness of ICIs in melanoma treatment. We employed multipanel markers to predict the response to immune checkpoint inhibitors by characterizing the gene expression signatures of skin immune cells in systemic lupus erythematosus (SLE), atopic dermatitis (AD), and psoriasis (PS). By analyzing single-cell RNA sequencing data from each dataset, T cell gene signatures from autoimmune skin diseases exhibit a complex immune response in tumors that responded to immunotherapy. Based on that CD86 and CD80 provide essential costimulatory signals for T cell activation, we observed that interaction of CD86 signaling has been enhanced in the T cells of patients with SLE, AD, and PS. Our analysis revealed a common increase in CD86 signals from dendritic cells (DCs) to T cells in patients with SLE, AD, and PS, confirming that dendritic cells produce pro-inflammatory cytokines to activate T cells. Thus, we hypothesize that T cell gene signatures from autoimmune skin diseases exhibit a pro-inflammatory response and have the potential to predict cancer immunotherapy. Our study demonstrated that T cell gene signatures derived from inflammatory skin diseases, particularly SLE and PS, hold promise as potential biomarkers for predicting the response to immune checkpoint blockade therapy in patients with melanoma. Our data provide an understanding of the immune-related characteristics and differential gene expression patterns in autoimmune skin diseases, which may represent promising targets for melanoma immunotherapy.

## Introduction

Skin homeostasis, a complex process regulated by the interplay among keratinocytes, fibroblasts, and immune cells, is crucial for maintaining a healthy skin environment^1, 2^. However, autoimmune skin diseases arise from immune system dysregulation, leading to the production of autoantibodies targeting self-antigens in the skin. This dysregulation leads to the development of systemic lupus erythematosus (SLE), atopic dermatitis (AD), and psoriasis (PS)^3–5^. These chronic inflammatory skin conditions are prevalent worldwide, can cause severe and persistent disease that can impact patients throughout their lives.

Systemic lupus erythematosus (SLE), is a complex autoimmune disease affecting multiple organs and systems, including the skin. It is characterized by the production of autoantibodies target various body components, leading to widespread inflammation^6^. Over time, SLE progresses and poses a life-threatening risk as it causes damage to major organs. Atopic dermatitis (AD), or eczema, is a chronic inflammatory skin condition characterized by intense itching, redness, dryness, and skin lesions. The immune dysregulation in atopic dermatitis involves an overactive T helper type2 (Th2) response^7^, leading to increased production of interleukins such as IL-4, IL-5, and IL-13^8^. This aberrant immune response results in skin barrier dysfunction, increased susceptibility to allergens, and chronic inflammation^9^. Psoriasis (PS) is a chronic autoimmune skin disorder characterized by the rapid turnover of skin cells, resulting in thick, scaly patches on the skin^10^. The immune dysregulation in psoriasis involves overactive T helper type 1 (Th1) and Th17 responses^11–13^, leading to the production of pro-inflammatory cytokines such as tumor necrosis factor-alpha (TNF-α), interferon-gamma (IFN-γ), and interleukin-17 (IL-17). These cytokines contribute to the proliferation of keratinocytes^12^ and the recruitment of immune cells^14^, leading to the characteristic plaques and inflammation observed in psoriasis.

At the intersection of autoimmunity and cancer immunology, the relationship between the anti-tumor response and autoimmune diseases has been suggested, but remains unclear^15, 16^. Given the diseases’ distinct symptoms and mechanisms, our study aimed to establish a connection between immune cells derived from skin-related autoimmune diseases and their potential applications in cancer treatment.

Immune checkpoint inhibitors have shown significant clinical improvement in melanoma and have become the most effective anti-cancer agents, even as the first-line treatment settings^17^. Currently the predictive value of PD-L1 expression in tumors for immunotherapy response is limited in sensitivity and specificity. Additionally, the assessment of immune checkpoint blockade-related gene signatures (PD-1, PD-L1, and CTLA-4)^18^, whether expressed in tumor or immune cells, does not fully encompass the intricate tumor microenvironment involved in the immunotherapy response. In this study, we examined gene signatures derived from autoimmune skin diseases to determine their potential utility in guiding immunotherapy in patients with melanoma.

## Methods

### Data sources and processing

**Figure 1:**
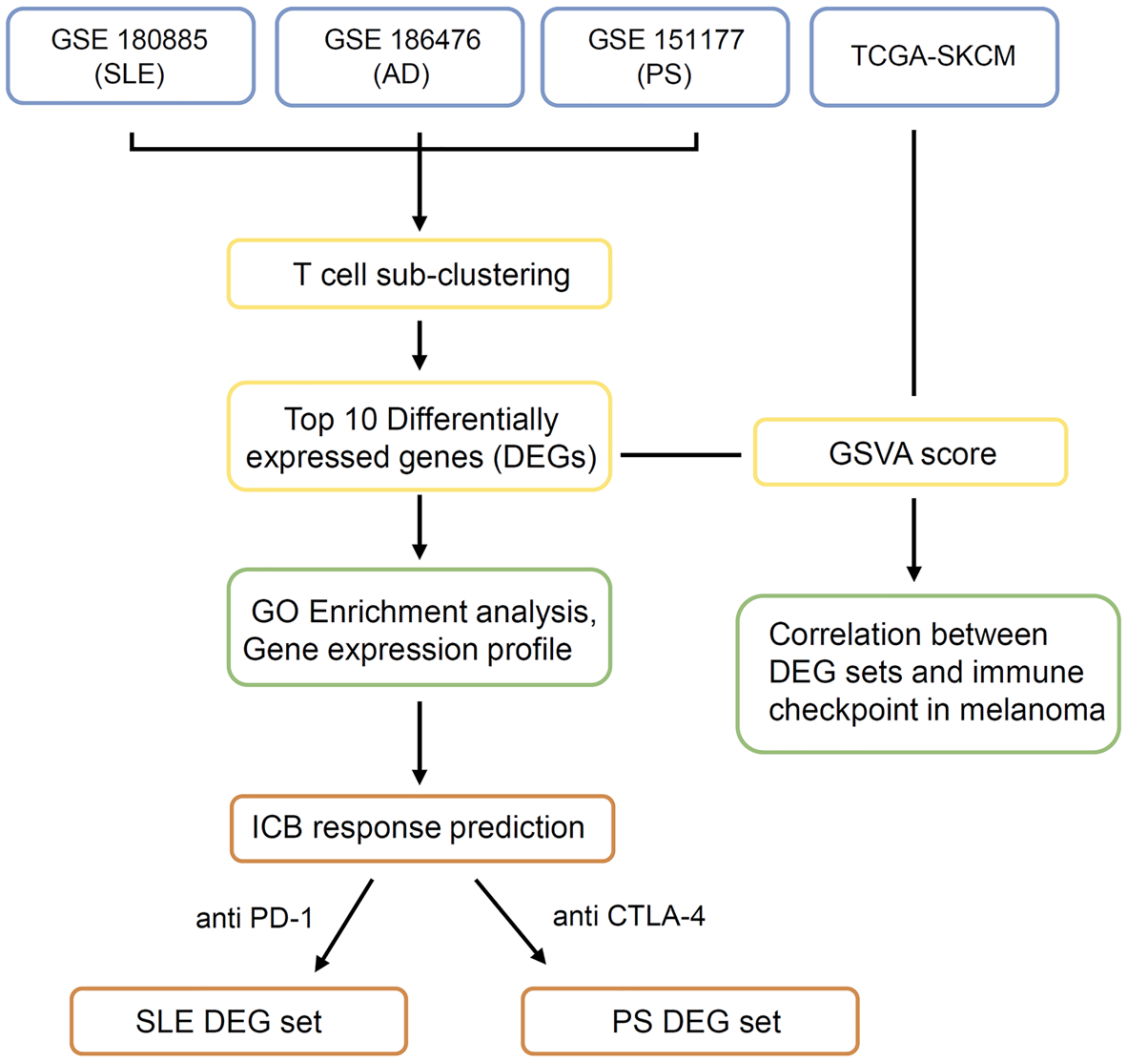
Overview of the study design for data analysis. Common changes in immune cell clusters were identified in the single-cell RNA sequencing data from patients with SLE, AD, and PS, confirming common immunological characteristics among autoimmune skin diseases. Pathway analysis and DEG analysis were performed through T cell sub-clustering of SLE, AD, and PS patients. Furthermore, we assessed the correlation between immune checkpoint genes in the TCGA-SKCM data. The potential of the T cell DEGs as a biomarker was examined through an ICB response prediction tool. (SLE: Systemic Lupus Erythematosus; AD: Atopic Dermatitis; PS: Psoriasis).

Figure 1 presents the workflow of the study. We analyzed three single-cell RNA sequencing datasets from the Gene Expression Omnibus (GEO; https://www.ncbi.nlm.nih.gov/gds/) database to examine the transcriptomic characteristics and cellular composition of autoimmune skin diseases. The study includes single-cell RNA sequencing data from GSE180885^19^, consisting of samples from healthy controls (n = 14) and SLE patients (n = 7). Additionally, data from GSE18476^20^ were used for Atopic Dermatitis, comprising healthy controls (n = 7) and AD patients (n = 8). Lastly, data from GSE151177^21^ were analyzed for psoriasis, including healthy controls (n = 6) and psoriasis patients (n = 13).

### Preprocessing and normalization of Datasets for analysis

The datasets were preprocessed using the R package Seurat (version 4.0.2) to eliminate low-quality cells using the following options: SLE datasets, 200 < nFeatures < 6000, nCount < 50,000, and percent.mt < 35; AD datasets, 200 < nFeatures < 6000, nCount < 40,000, and percent.mt < 70; and PS datasets, 100 < nFeatures < 5000, nCount < 40,000, and percent.mt < 25. The filtered counts were normalized using the SCTransform^22^ function, which included mitochondrial and ribosomal gene percentages regression. We applied canonical correlation analysis (CCA) using the FindIntegrationAnchors and IntegrateData commands in Seurat to correct batch effects. After integration, the counts were log-normalized using the NormalizeData function and scaled using ScaleData with the default settings. The dimensional reduction was performed using the RunUMAP function, with the first 30 principal components determined from the elbow plot. Clustering was perfomed using the FindNeighbors and FindClusters functions with a resolution of 0.6.

### Integrate and harmonize single-cell data for comprehensive analysis

SLE, AD, and PS Seurat object was integrated using the Seurat R package^23^. Integration anchors were computed based on consistently variable features across all datasets, and the IntegrateData function was used to merge the Seurat datasets using these computed anchors. The Harmony R package (version 0.1.0)^24^ integrate variances from different data sources and generated harmony embeddings to address batch effects. Clustering is subsequently performed using the updated harmony embeddings. Consequently, the integrated SLE Seurat object comprised 9834 cells, with an average of 23,286 genes detected per cell. Similarly, the AD Seurat object comprised of 20,534 cells, with an average of 9591 genes detected per cell. The PS Seurat object also included 11,113 cells, with an average of 22,723 genes detected per cell.

### Identification of cluster markers and cell type annotation

We employed the Seurat FindAllMarkers function to identify the cluster markers and performed a Wilcoxon rank-sum test. Genes with a logFC value greater than 0.25 and a false discovery rate (FDR) lower than 0.05 were considered as cluster markers. Annotation of cell types for each cluster was conducted manually by comparing the selected cluster markers with the reference genes. The reference genes used to determine the clusters can be found in (Fig. S1).

### Differential Gene Expression Analysis and Pathway Enrichment

Differential gene expression was analyzed using the Seurat FindMarkers function with the default Wilcoxon rank-sum test. The integrated Seurat object was divided into cell type-specific objects, and differential expression analysis identified significant genes with a logFC > 0.15 and *p* < 0.01.

Pathway analysis was performed using the Enrichr (3.0) R package with phenotype-based permutation tests and specific pathway databases, such as the 2021 Gene Ontology (GO) molecular function and GO biological process^25^. Significantly enriched pathways (*p* < 0.05) were considered statistically significant and were selected for further analysis.

### GSVA

The GSVA (V1.38.2) software package, available from R/Bioconductor, was used for this study serving as a non-parametric, unsupervised method for estimating variation in pre-defined gene sets in TCGA bulk RNA-seq datasets^26^. The inputs for the GSVA algorithm comprised a gene expression matrix comprising log2 normalized RSEM values and pre-defined gene sets specific to the SLE, AD, and PS datasets. GSVA scores were computed using a Kolmogorov-Smirnoff-like random walk statistic, and a negative value was assigned to a particular sample and gene set.

### Cellular communication network

Interactions between cell clusters were analyzed using the CellChat R package (version 1.1.3) and CellChatDB reference database^18^. The probability of communication for specific signaling pathways was computed based on each cell group’s upregulated and downregulated ligand-receptor genes. The netVisual_aggregate function was used to visualize the number and strength of interactions between the cell groups in circular plots. Significant signaling pathways were ranked using the rankNet function, which measure the differences in the overall information flow between the two conditions.

## Results

### Characterizing immune cells in autoimmune skin diseases analyzed through single-cell RNA seq

**Figure 2:**
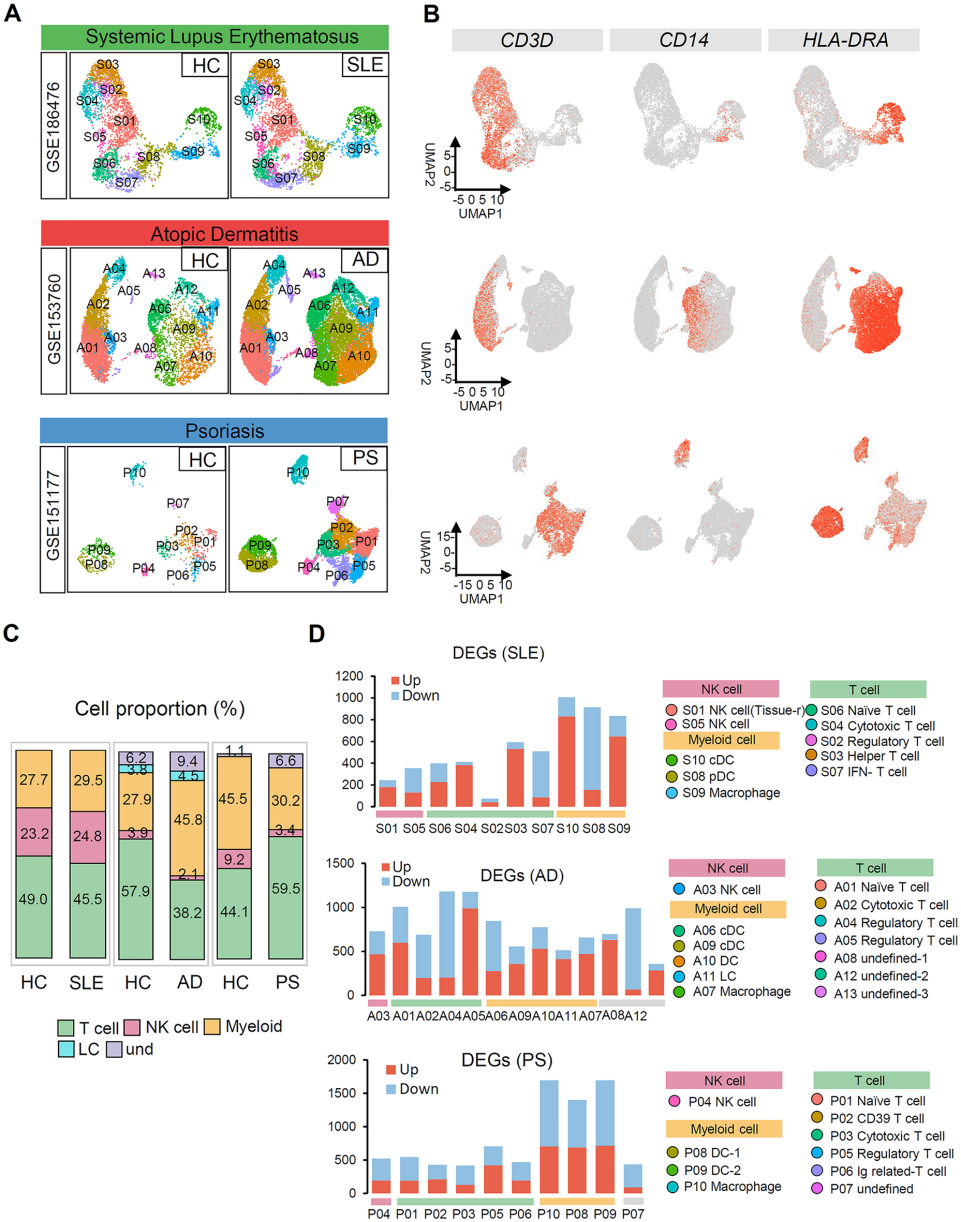
Immune-related characteristics of autoimmune skin diseases analyzed through single-cell RNA seq. (**A**) Uniform manifold approximation and projection (UMAP) of all cells that passed quality control. (SLE: n = 7419cells; AD: n = 23,234cells; PS: n = 10,214 cells). (**B**) Normalized expression levels of marker genes for each immune cell type on UMAP plots. CD3D (T cells), CD14(Monocytes/Macrophages), HLA-DRA (Dendritic Cells). (**C**) Stack bar graph showing the proportion of immune cell cluster in each SLE, AD, PS datasets. (**D**) Bar chart showing up/down regulated DEGs in each cluster. Each cluster is annotated into a T cell, NK cell, and Myeloid cell by its own marker genes.

To investigate the immune cell subpopulations associated with autoimmune skin diseases, we analyzed a dataset including SLE, AD, and PS through single-cell RNA seq. Focusing specifically on the immune cells, we compared the characteristics of SLE, AD, and PS. This sub-clustering of immune cells from each dataset was based on the expression of PTPRC (CD45) (Fig. S1B). Visualization using uniform manifold approximation and projection (UMAP) revealed ten distinct clusters consisting of CD8^+^ T cells, natural killer (NK) cells, macrophages, and dendritic cells (DCs) in both systemic lupus erythematosus (SLE) and healthy control skin immune cell datasets. Patients with atopic dermatitis (AD) exhibit a complex and diverse inflammatory profile involving, CD8^+^ T cells, NK cells, dendritic cells, and Langerhans cells (LCs)^27^. Similarly, in the psoriasis (PS) dataset, our analysis revealed the presence of ten clusters encompassing CD8^+^ T cells, CD4^+^ T cells, NK cells, and dendritic cells. We examined the expression levels of immune cell markers within each cluster to validate each individual cell cluster (Fig. 2A, B, and S2A). Distinct transcriptomic profiles were observed among well-annotated cell types, evident from the differential gene expression patterns observed in the heatmap for each cluster.

Next, we calculated the proportion of cells within the immune cell clusters in the SLE, AD, and PS datasets (Fig. 2C). Although no significant differences were observed in SLE, distinct variations were observed in the proportions of T and myeloid cells in the AD and PS datasets. Specifically, patients with AD showed a decrease in the T cell proportion and an increase in the myeloid cell proportion, whereas patients with PS displayed an increase in the T cell proportion and a decrease in the myeloid cell proportion. Furthermore, we analyzed the transcriptomic profiles and identified 620 differentially expressed genes (DEGs) in the SLE datasets, with 372 up-regulated and 248 down-regulated genes showing statistical significance (adjusted *p* < 0.01; log2foldchange [FC] < 0 or > 0). Similarly, the AD dataset revealed 773 DEGs (551 up-regulated and 222 down-regulated), whereas the PS dataset contained 765 DEGs (329 up-regulated and 436 down-regulated) (Fig. 2D). Notably, significant changes in T cell and myeloid cell cluster DEGs showed significant changes in patients with SLE and AD. Overall, these findings provide valuable insights into immune cell proportions and differential gene expression in autoimmune skin diseases, shedding light on their underlying mechanisms.

### Interactions between T cells and Myeloid cells in autoimmune skin diseases

**Figure 3:**
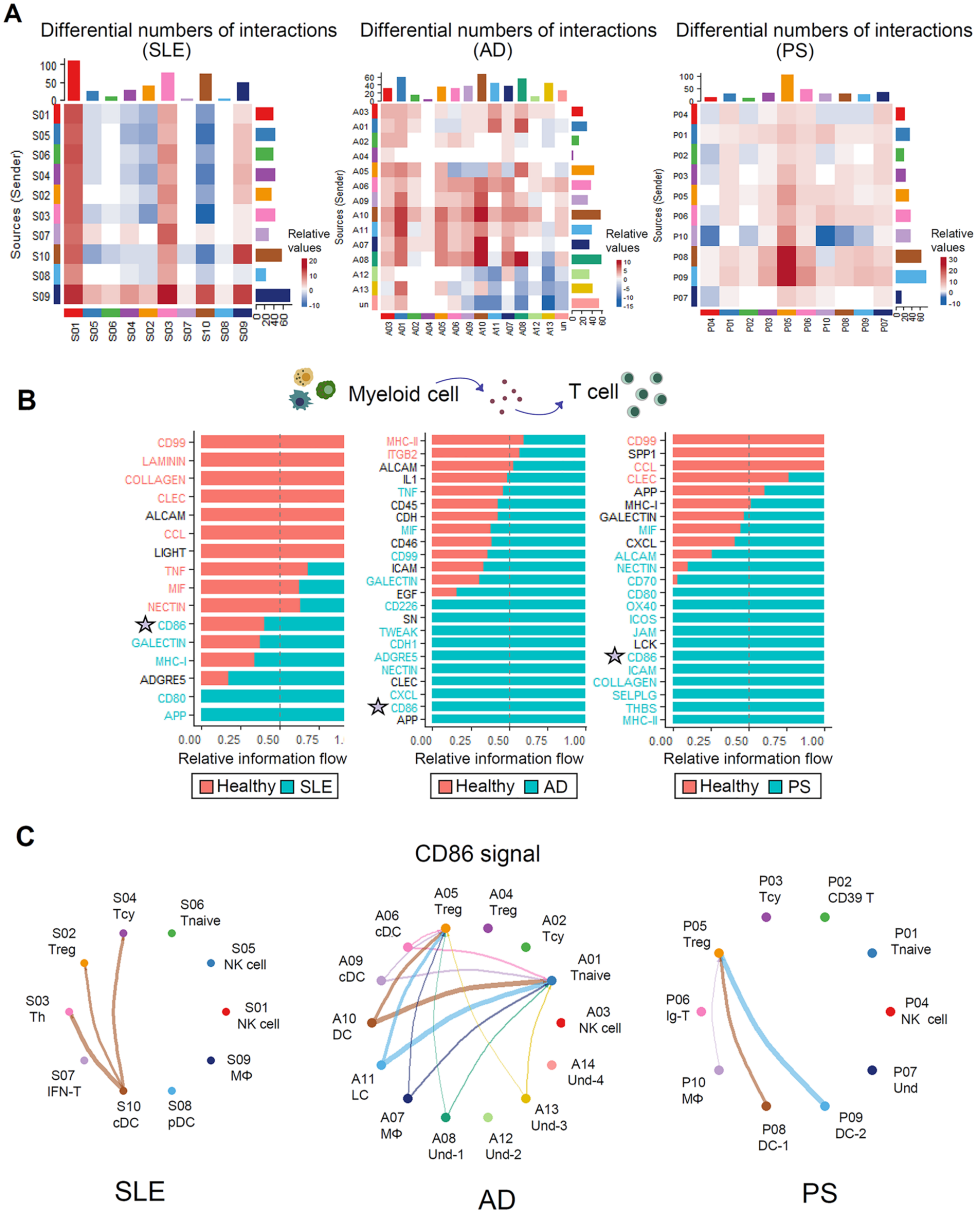
Investigate cell–cell signaling from myeloid cells to T cells in autoimmune skin diseases. (**A**) Heatmap shows the differential numbers of interactions between SLE, AD, PS, and HC. In the color bar, red indicates increased signaling in the second dataset compared to the first, while blue indicates decreased signaling. (**B**) Bar graphs showing the ranking of major signals from myeloid cells to T cells in SLE, AD, and PS datasets. (**C**) The circle plot visualizes the cell-to-cell network, highlighting the increased outgoing signals of DCs in SLE, AD, and PS datasets. The direction of the signals is indicated by arrows and edge color, with thicker edges representing a higher sum of weight key signals between populations.

Given the significant changes observed in T and myeloid cells in patients with SLE, AD, and PS, we focused on investigating their interactions using CellChat analysis. This analysis highlighted the prominent differences in the potential interactions between T cells and myeloid cells (Fig. 3A and S3A).

Specifically, we observed a standard increase in CD86 signals from myeloid cells to T cells in the SLE, AD, and PS datasets^28, 29^ (Fig. 3B). The interaction between CD86 (B7-2) ligands, CD80 (B7-1), and CD28 forms a well-characterized pathway^30^. CD86, and CD80, deliver essential costimulatory signals for T cell activation and survival. Moreover, CD80/CD86 exhibited a higher affinity for CTLA-4 on activated T cells than CD28, thereby contributing to regulating the T cell response^31^. In patients with SLE, CD86 signals were observed to flow from the cDCs (Conventional dendritic cell) to T helper cells, regulatory T cells, and cytotoxic T cells^32^ (Fig. 3C). Similarly, signals from cDCs^33^, LCs^34^, and macrophages targeted naïve T cells and regulatory T cells in AD patients. In patients with PS, DCs and macrophages have been identified as sources of signals that flow into regulatory T cells ^35^. Signals from T cells flowing into myeloid cells as a source were not observed in patients with SLE, AD, and PS. (Fig. S3B). Based on these findings, we investigated T cell changes through sub-clustering analysis within the SLE, AD, and PS immune cell populations.

### Up-regulated pathways and key genes in T cells of autoimmune skin diseases

**Figure 4:**
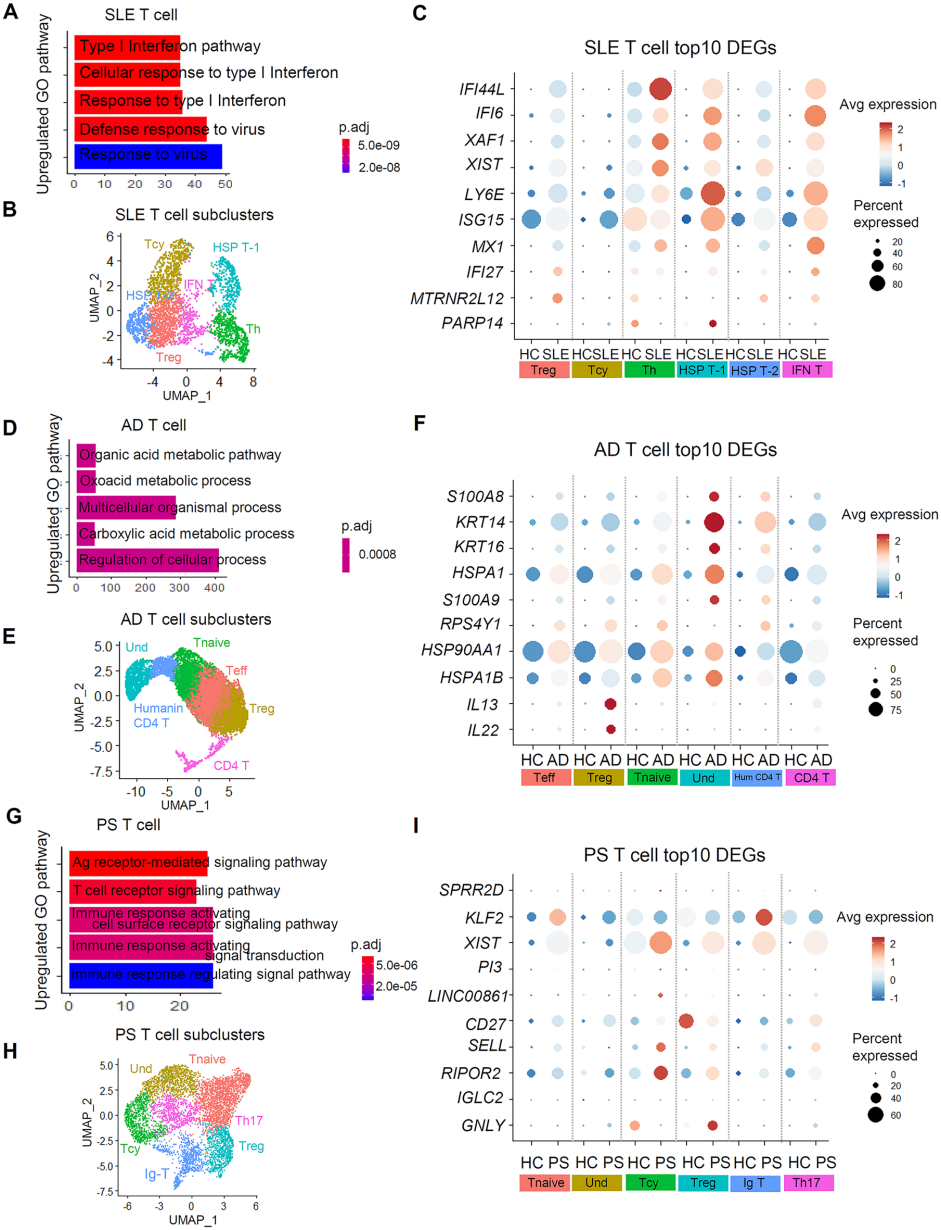
T cell gene signatures derived from autoimmune skin diseases exhibit a pro-inflammatory response. (**A**, **D**, **G**) GO enrichment analysis for upregulated pathway in SLE, AD, PS datasets. The p-value adjusted by Bonferroni correction is indicated by color. (**B**, **E**, **H**) Cells are colored based on T cell type. (**C**, **F**, **I**) Top 10 differentially expressed genes ranked by foldchange SLE, AD, PS T cells compared to HC. Treg, regulatory T cell; Tc, cytotoxic T cell; Th, helper T cell; HSP T, heat shock protein-related T cell; IFN T, Interferon related T; Tnaive, naïve T cell; Hum CD4 T, humanin CD4T; Ig T, Immunoglobulin related T.

Pathway analysis using Enrichr revealed distinct molecular pathways associated with each disease. Type I IFN pathways were upregulated in T cells in patients with SLE, consistent with previous findings linking Type I IFNs to regulatory T cell dysfunction in SLE^36, 37^ (Fig. 4A and S4A). Also, elevated IFN levels in SLE patients are linked to enhanced T cell activation by DCs^38^. We aimed to identify genes within the T cell cluster that were highly ranked in SLE patients (Fig. 4B, C, and S4B). The top 10 differentially expressed genes (DEGs) in SLE T cells included IFI44L, IFI6, XAF1, XIST, LY6E, ISG15, MX1, IFI27, MTRNR2L12, and PARP14^39, 40^. Regulatory T cell play a crucial role in controlling autoimmune skin diseases, including AD^41^. In patients with AD, the top 10 DEGs in T cells included S100A8, KRT14, KRT16, HSPA1, S100A9, RPS4Y1, HSP90AA1, HSPA1B, IL13, and IL22^42^ (Fig. 4D–F, S4C, and S4D). In patients with PS, T cells exhibit upregulation of the T cell receptor signaling and immune response activation pathways (Fig. 4G). The top 10 DEGs in PS T cells comprised SPRR2D, KLF2, XIST, PI3, LINC00861, CD27, SELL, RIPOR2, IGLC2, and GNLY^43^ (Fig. 4H,I, S4E, and S4F). These findings provide valuable insights through pathway analysis and specific genes associated with immune cell function in each autoimmune skin disease.

### Association between expression of inflammatory skin disease and immune response in cancer

T cells from skin-derived inflammatory diseases exhibit differential gene expression patterns, including genes known to be involved in immune activation in cancer^44^. Specifically, in tumors, IFN-related genes are recognized to activate T cells and play a crucial role in tumor infiltration. Building on this knowledge, we hypothesized that these differential gene expression patterns in inflammatory skin disease T cells are associated with specific immunological programs in cancer. Additionally, acknowledging cancer’s impact on body balance via neuroendocrine pathways, as seen in skin cancers, we uncovered connections between immune responses in skin-related diseases and cancer^45, 46^. To investigate this hypothesis, we focused on melanoma, a type of skin cancer, and evaluated the relationship between T cell DEGs from autoimmune skin diseases and immune checkpoint genes.

**Figure 5:**
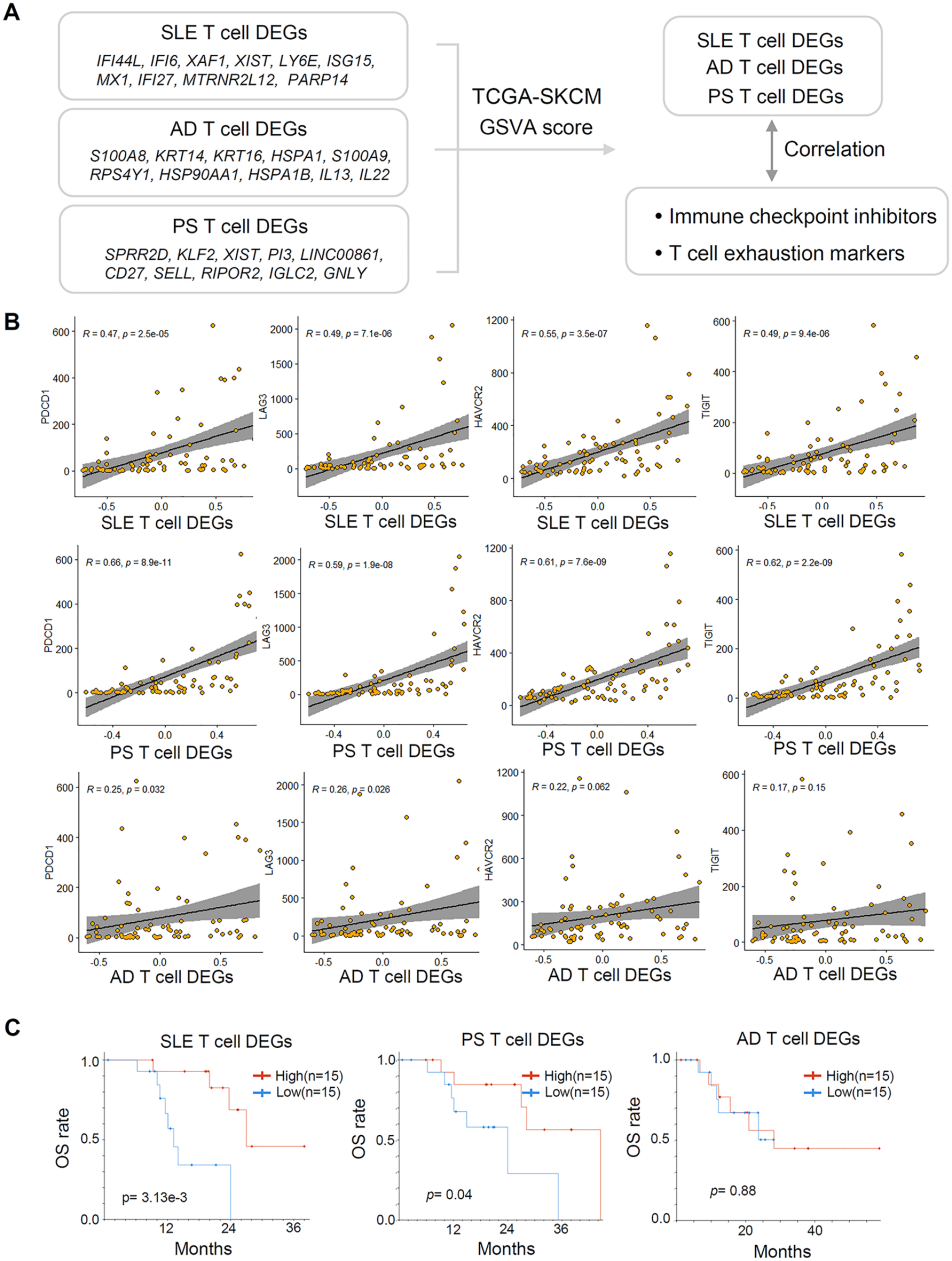
SLE and PS T cell DEGs show positive correlations with immune checkpoint inhibitor genes in melanoma. (**A**) A Simple scheme of the workflow. Using each top T cell DEGs as a set, run GSVA in the TCGA-SKCM expression matrix. (**B**) Spearman correlation of SLE, AD, and PS T cell DEGs GSVA score with expression of immune checkpoint inhibitors and T cell exhaustion markers; PDCD1 (PD-1), LAG3, HAVCR2 (TIM-3), TIGIT (**C**) Kaplan–Meier plot for SLE T cell DEGs set-high group (n = 15, 20%) and SLE T cell DEGs set-low group (n = 15, 20%) in TCGA SKCM cohort patients. Logrank *p* = 3.13e^-3^; PS- DEGs set-high group (n = 15, 20%) and PS T cell DEGs set-low group (n = 15, 20%), Logrank *p* = 0.04; AD-DEGs set-high group (n = 15, 20%) and AD T cell DEGs set-low group (n = 15, 20%), *p* = 0.88.

We aimed to determine whether these skin disease T cell DEGs could predict the response to immune checkpoint therapy in patients with melanoma by examining their correlations with immune checkpoint gene response gene expression signatures in the TCGA SKCM dataset (Fig. 5A). Through the analysis of T cell DEGs GSVA enrichment scores, we compared the expression levels of immune checkpoint genes (PD-L1, PDCD1, and CTLA-4)^47, 48^ and T cell exhaustion markers (TIM3, LAG3, HAVCR2, and TIGIT)^49^ with the differential gene expression patterns of SLE, AD, and PS in the TCGA-SKCM dataset. High PD-L1 expression in tumors has been associated with improved clinical outcomes following PD-1 blockade therapies, although responders have not been fully stratified^50, 51^. Our results demonstrated significant correlations between the T cell differential gene expression patterns of SLE and PS patients and the ICB marker PD1, as well as exhaustion markers LAG3, TIGIT, and HAVCR, indicating their potential to predict responders and non-responders. However, patients with AD did not show any significant correlations with these markers (Fig. 5B and S5A).

Additionally, we assessed overall survival (OS) rates by categorizing melanoma patients into T cell DEGs set-high and T cell DEGs set-low expression groups (Fig. 5C). Interestingly, melanoma patients with high expression of SLE and PS T cell DEGs exhibited increased survival rates. These findings suggest that differential T cell gene expression patterns associated with inflammatory skin diseases could be markers for distinguishing patients who may benefit from immunotherapy.

### Potential ICB response prediction biomarkers derived from SLE and PS T cell DEGs

Based on these findings, this study explores the potential of the identified DEGs as predictive tools for response to immune checkpoint blockade therapy. To evaluate their potential, we employed the Tumor Immune Dysfunction and Exclusion (TIDE) algorithm and focused on the top 10 T cell DEGs in patients with SLE, AD, and PS as novel biomarkers. By comparing their predictive capabilities with existing biomarkers, we found that the T cell DEGs in SLE exhibited superior predictive value compared to tumor mutational burden (TMB), MSI score, T clonality, and B clonality. Notably, among the ICB subcohorts, the SLE T cell DEGs set exhibited an area under the curve (AUC) greater than 0.5 in 15 out of the 22 (66%) subcohorts, which was comparable to the predictive performance achieved by TIDE (17 out of the 25, 68%). Additionally, 59% (13 of 22) of the subcohorts consisted of patients with PS, while 50% (11 of 22) consisted of patients with AD, both showing an AUC greater than 0.5 (Fig. S6A).

**Figure 6:**
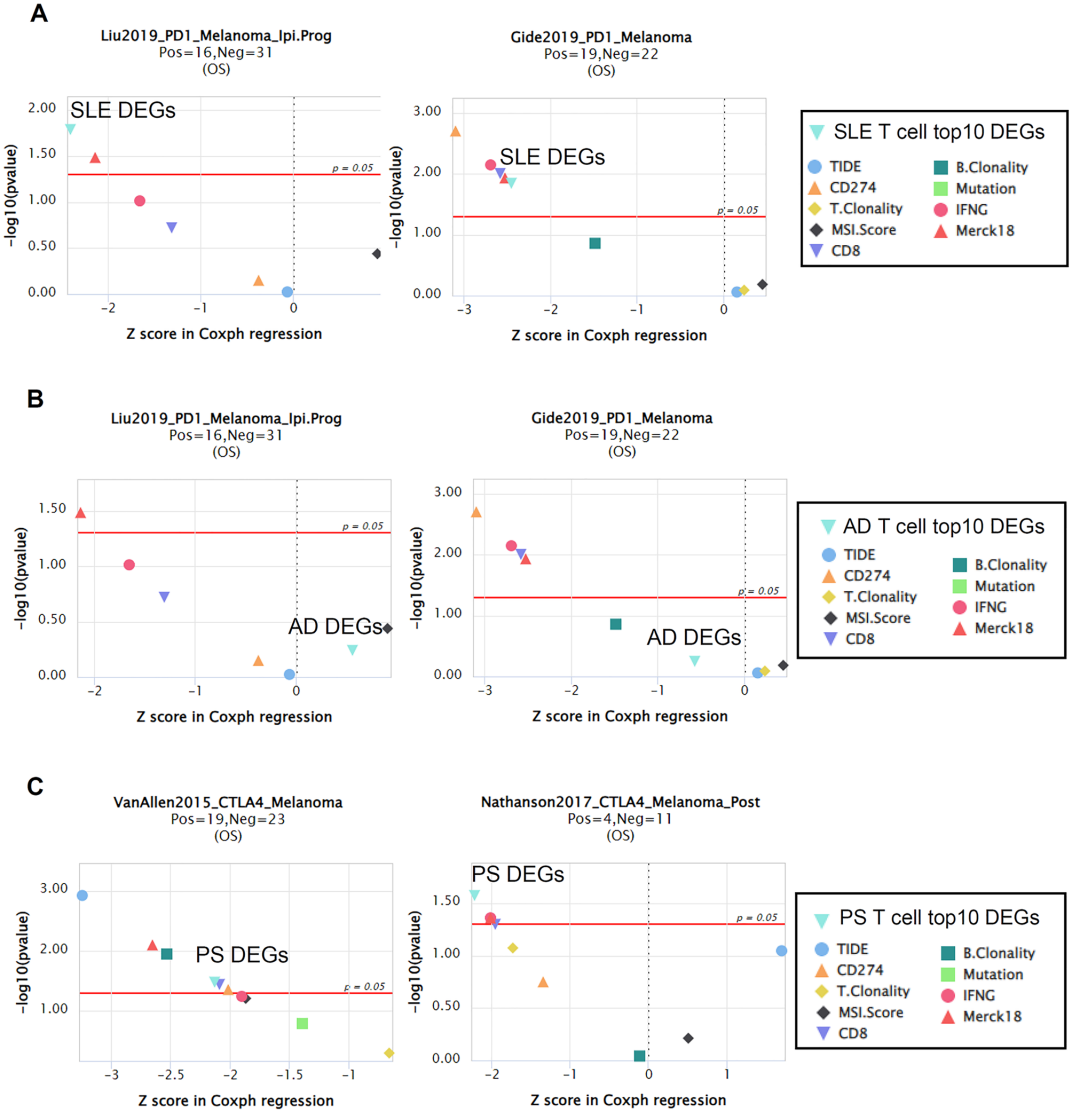
Evaluation of SLE and PS T cell DEGs as biomarkers for ICB response prediction. (**A**) Comparison of SLE DEGs expression with other biomarkers in two anti-PD-1 responder melanoma patient data sets. (**B**) Comparison of AD DEGs expression with other biomarkers in two anti-PD-1 responder melanoma patient data sets. (**C**) Comparison of PS DEGs expression with other biomarkers in two anti-CTLA-4 responder melanoma patient data sets.

Furthermore, our analysis demonstrated that SLE T cell DEGs set effectively predicted patient response in two melanoma cohorts treated with anti-PD1 therapy (Liu2019_PD1_Melanoma and Gide2019_PD1_Melanoma) (Fig. 6A). Similarly, the PS T cell DEGs set showed a predictive value for patient response in three melanoma cohorts treated with anti-PD1 therapy (Liu2019_PD1_Melanoma) and anti-CTLA4 therapy (VanAllen2015_CTLA4_Melanoma and Nathanson2017_CTLA4_Melanoma_Post) (Fig. 6C, and S6B). However, no significant difference was observed in the AD T cell DEGs set among the melanoma cohorts treated with either anti-PD1 or anti-CTLA4 therapy (Fig. 6B). These findings, and previous data support the potential utility of T cell gene signatures derived from inflammatory skin diseases as valuable tools for identifying patients with melanoma who could benefit from immune checkpoint blockade therapy. These data demonstrate that the T cell gene signature of patients with SLE can be a marker for distinguishing responders to PD-1 therapy, whereas the T cell gene signature of patients with PS can serve as a marker for distinguishing responders to CTLA-4 therapy.

## Discussion

This study observed that immune cells enriched in patients with autoimmune skin diseases could be predictive markers for the clinical response to anti-PD1 and anti-CTLA4 therapy. This is the first study to analyze and compare T cell function in patients with SLE, AD, and PS, demonstrating their potential as markers for immunotherapy in patients with melanoma. The diverse gene signatures associated with inflammatory skin diseases suggest a complex immune response in tumors that respond to immunotherapy. These findings suggest the potential of using skin-derived immune cell profiles as biomarkers to guide immunotherapy in patients with melanoma.

We observed increased interactions between T cells and myeloid cells in SLE, AD, and PS patients. To further investigate these interactions, we examined the differences between myeloid cells and T cells in these diseases. Specifically, our analysis revealed an expected increase in CD86 signaling from dendritic cells to T cells in patients with SLE, AD, and PS patients, indicating its potential role in immune activation.

CD86, and CD80, provides costimulatory signals for T cell activation and survival. CD80/CD86 exhibited a higher affinity for CTLA-4 on activated T cells than CD28, suggesting its involvement in regulating the T cell response. Dendritic cells, known to express CD86 among other costimulatory molecules, regulate T cell activity through CTLA4^52^. The increased association between dendritic cells and T cells via CD86 signaling prompted us to explore immune activation in cancer. Therefore, we focused on melanoma and evaluated the relationship between differential gene expression patterns of SLE, AD, PS, and immune check-point genes. By considering the top 10 DEGs in the T cell clusters in patients with SLE, AD, and PS, we examined their correlation in patients with melanoma. Elevated PD-L1 expression in tumors enhances clinical outcomes in response to PD-1 blockade therapy. We confirmed its association with TCGA melanoma through enrichment score analysis of the autoimmune skin-disease T cell gene sets.

Furthermore, we observed that higher expression of the T cell DEGs set in patients with SLE and PS was associated with increased overall survival rates. These results shed light on the potential role of the skin-derived T cell DEGs set in predicting response to immune checkpoint blockade in patients with melanoma.

Our findings demonstrated that the SLE T cell DEGs set could effectively distinguish PD1 therapy responders, whereas the PS T cell DEGs set can identify CTLA4 therapy responders. However, the AD T cell DEGs set was unsuitable for distinguishing between these markers. Our findings revealed a significant increase in IFN-related genes in the SLE T cell DEGs set. It is well-established that IFN- γ plays a crucial role in inducing upregulation of PD-L1 and PD-L2 expression^53^. Furthermore, our investigation into the interaction between myeloid cells and T cells in patients with PS revealed an elevated ICOS signal and an increased CD86 signal in myeloid cells. ICOS serves as a co-stimulatory molecule for both CTLA-4 and CD28^54^. These insights explain the different associations between the DEGs and distinct immune checkpoint blockade pathways.

This study enhances our understanding of the complex interplay between immune cells and immune checkpoint pathways by unraveling the underlying mechanisms and molecular interactions. Identifying disease-specific T cell populations contribute to our understanding of disease mechanisms and uncovers promising targets for melanoma immunotherapy. These findings support the potential utility of disease specific DEGs as predictive biomarkers to guide the selection of appropriate immune checkpoint blockade therapies for patients with melanoma. Overall, the T cell gene signatures derived from inflammatory skin diseases, particularly SLE and PS, exhibit strong potential as biomarkers for predicting immune checkpoint blockade therapy in melanoma.

### Supplementary Information

Supplementary Figures.

## Data Availability

The datasets analyzed in this study are available in the following open access repositories; GEO, http://ncbi.nlm.nih.gov/geo/ (GEO: GSE186476, GSE153760, GSE151177); and TIDE, http://tide.dfci.harvard.edu/; The normalized tumor sample RNA sequencing data expression patterns from TCGA Skin cutaneous melanoma were downloaded from cBioportal. All code has been deposited on GitHub (https://github.com/kyu-hye/autoimmune-melanoma-icb-code.git ).
