# Supplementary material for: Gene signature from cutaneous autoimmune diseases provides potential immunotherapy-relevant biomarkers in melanoma
Source: Sci Rep. 2023 Sep 12;13:15023. doi: 10.1038/s41598-023-42238-3 (PMC10497583; doi:10.1038/s41598-023-42238-3)
Supplement: Supplementary file 1 — Supplementary Figures. [file 41598_2023_42238_MOESM1_ESM.docx]

**Supplemental file**


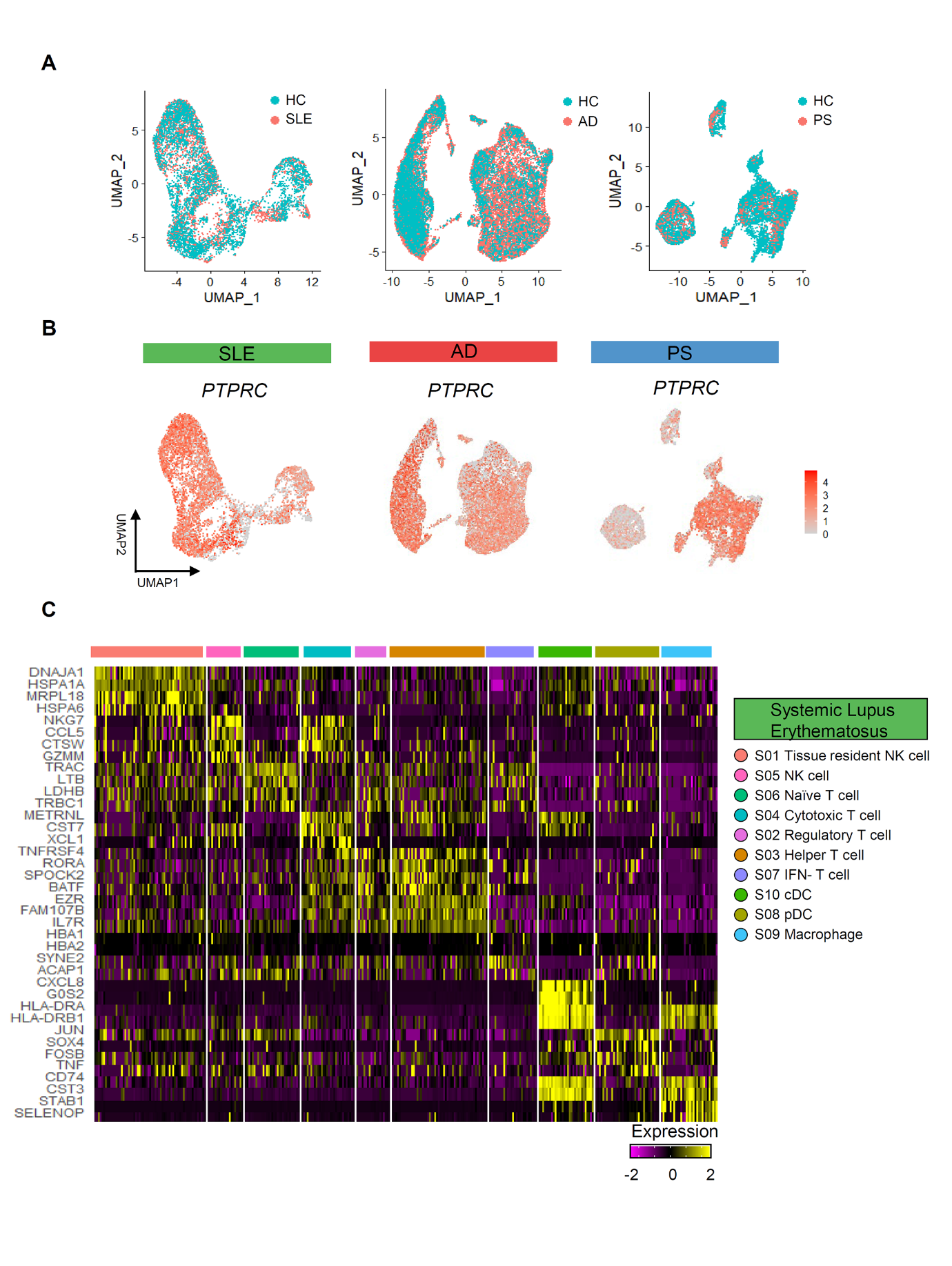


Supplementary Figure 1.

(A) Uniform manifold approximation and projection (UMAP) plot shows two groups of SLE and healthy controls; AD and healthy control; PS and healthy control. The two distinct groups are colored in blue and red, representing Control and SLE, AD, and PS patients, respectively.

(B) Normalized expression levels of PTPRC(CD45) for representing only immune cells are analyzed on UMAP plots.

(C) Heatmap displays the scaled expression of the top 4 cell markers ranked by fold change, in systemic lupus erythematosus dataset.
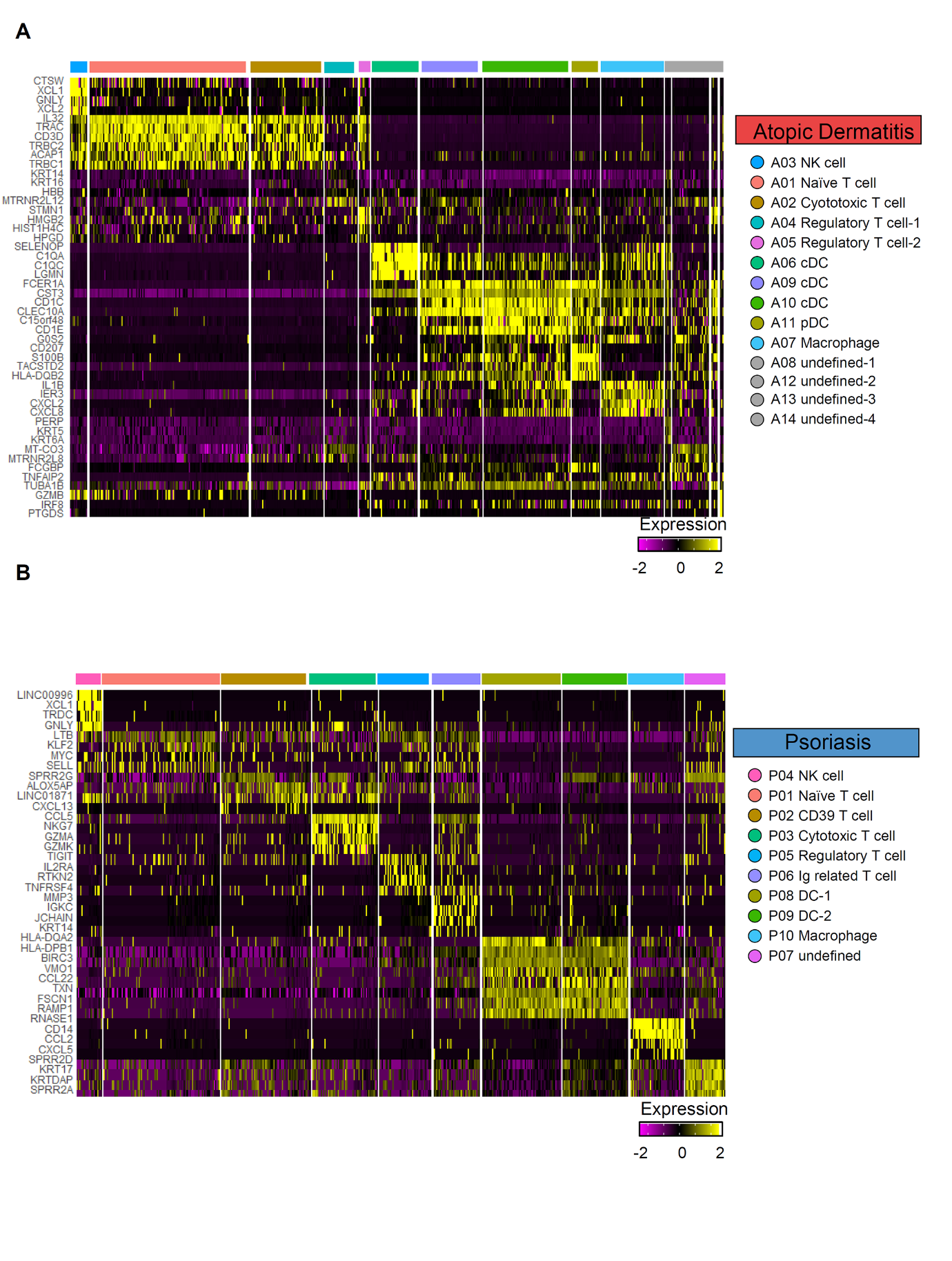


Supplementary Figure2.

(A, B) Heatmap displays the scaled expression of the top cell markers ranked by fold change, in atopic dermatitis, psoriasis dataset.


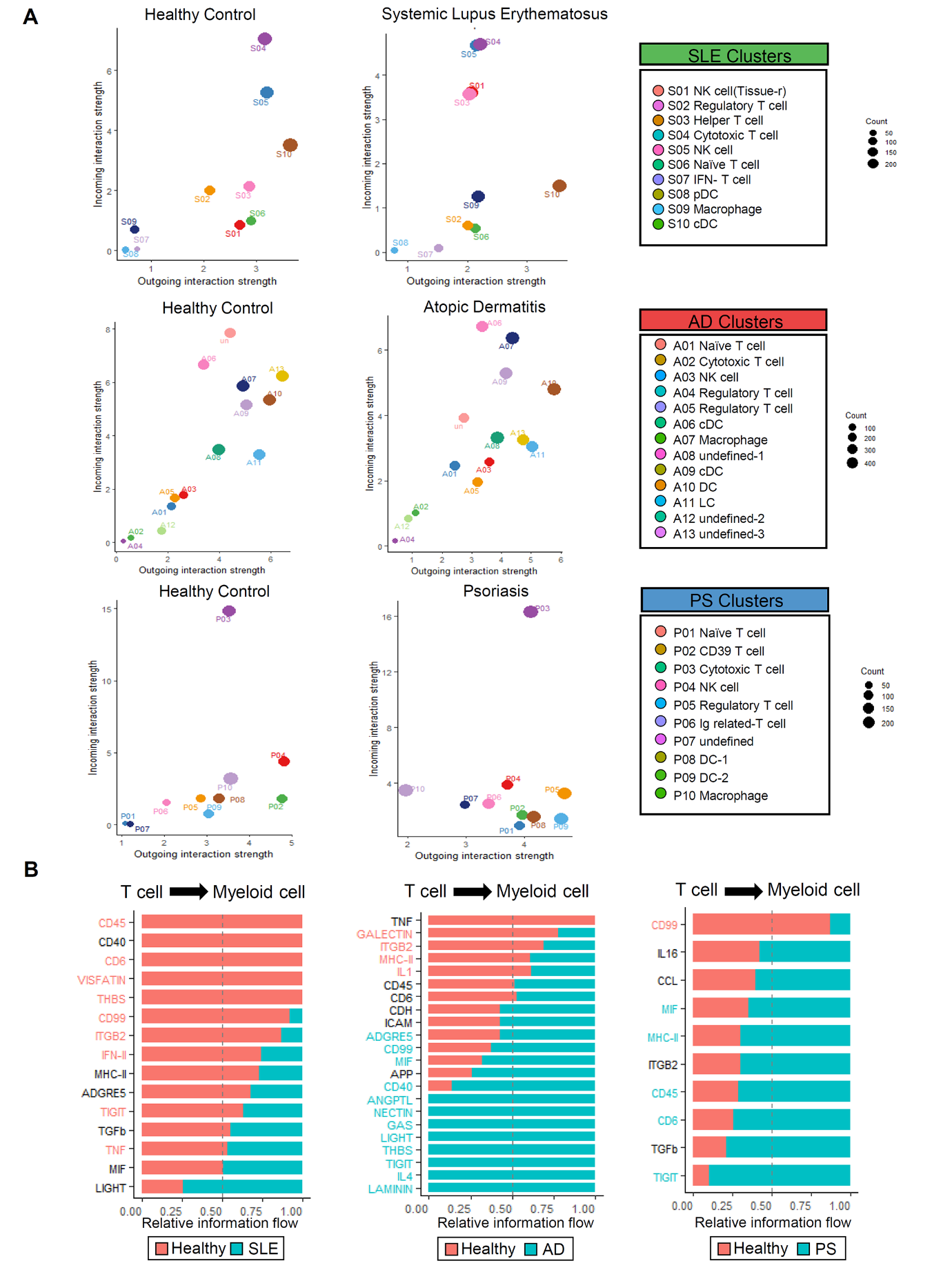


Supplementary Figure 3.

(A) Check the senders(sources) and receivers(targets) using a scatter plot for each SLE, AD, and PS datasets.

(B) Bar graphs showing the ranking of significant signals from T cells to myeloid cells in SLE, AD, and PS datasets.


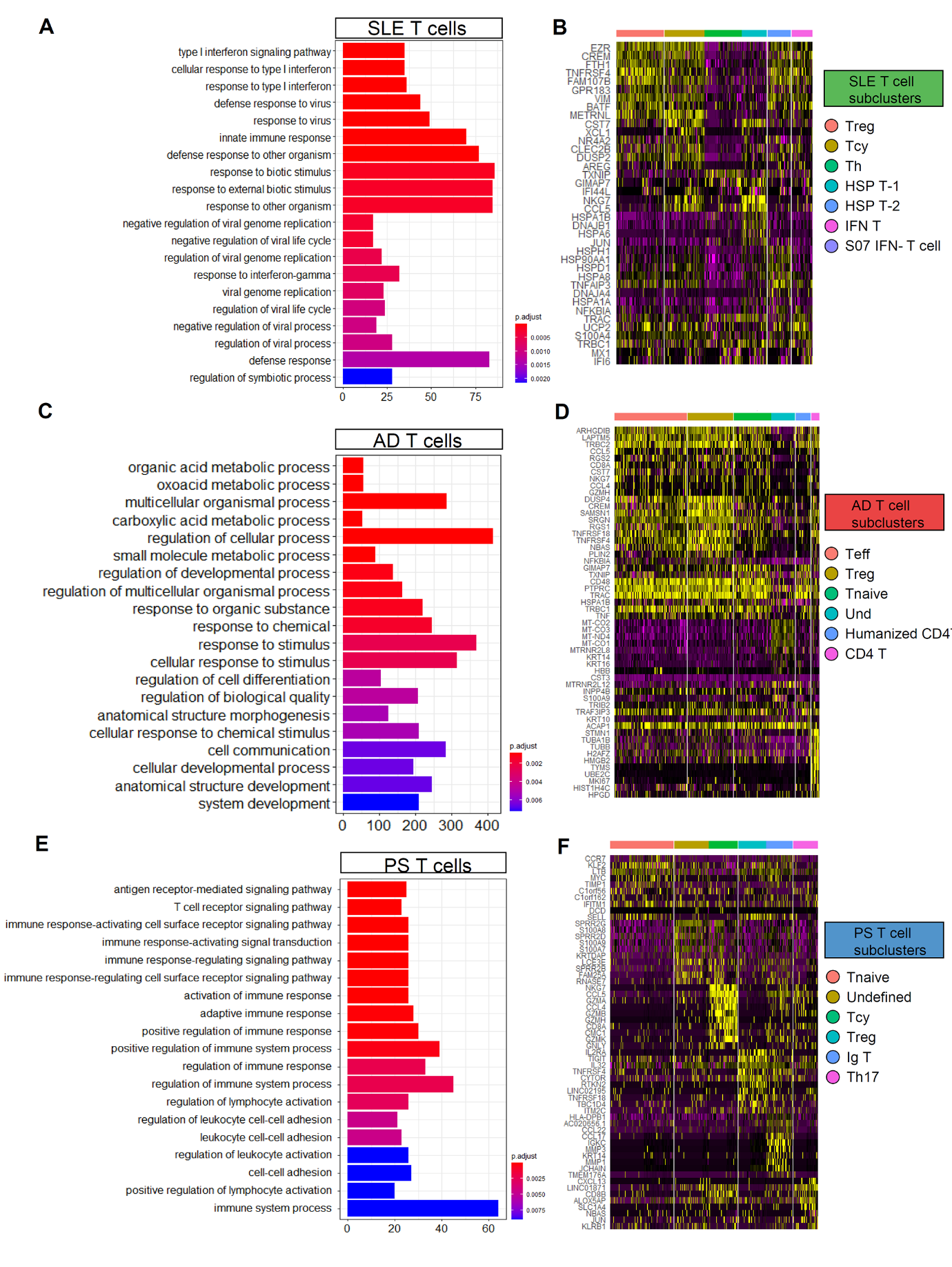
Supplementary Figure 4.

(A) Enriched Gene Ontology terms for the molecular signature for the SLE T cell subpopulation are displayed. Molecular signature ontology terms for each subpopulation are presented, with an adjusted *p* < 0.01, determined by the hypergeometric test.

(B, D, F) Unique expression profiles of the five T cell subpopulations observed in each dataset.

(C, E) GO terms for molecular signature for AD and PS T cell subpopulation.


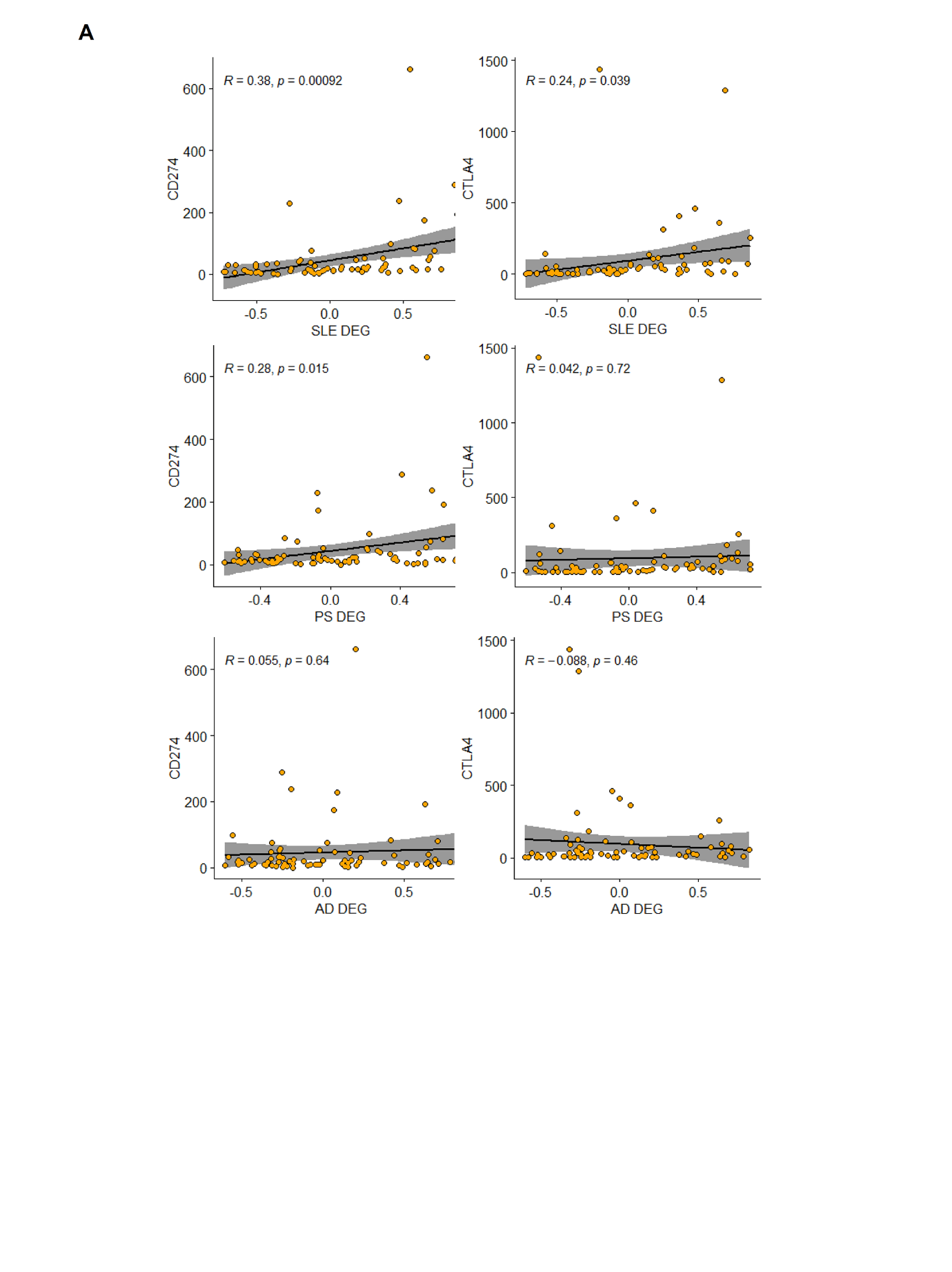


Supplementary Figure 5.

(A) Spearman correlation of SLE, AD, and PS T cell DEGs set GSVA score with expression of immune checkpoint inhibitors; CD274 (PD-L1), CTLA-4


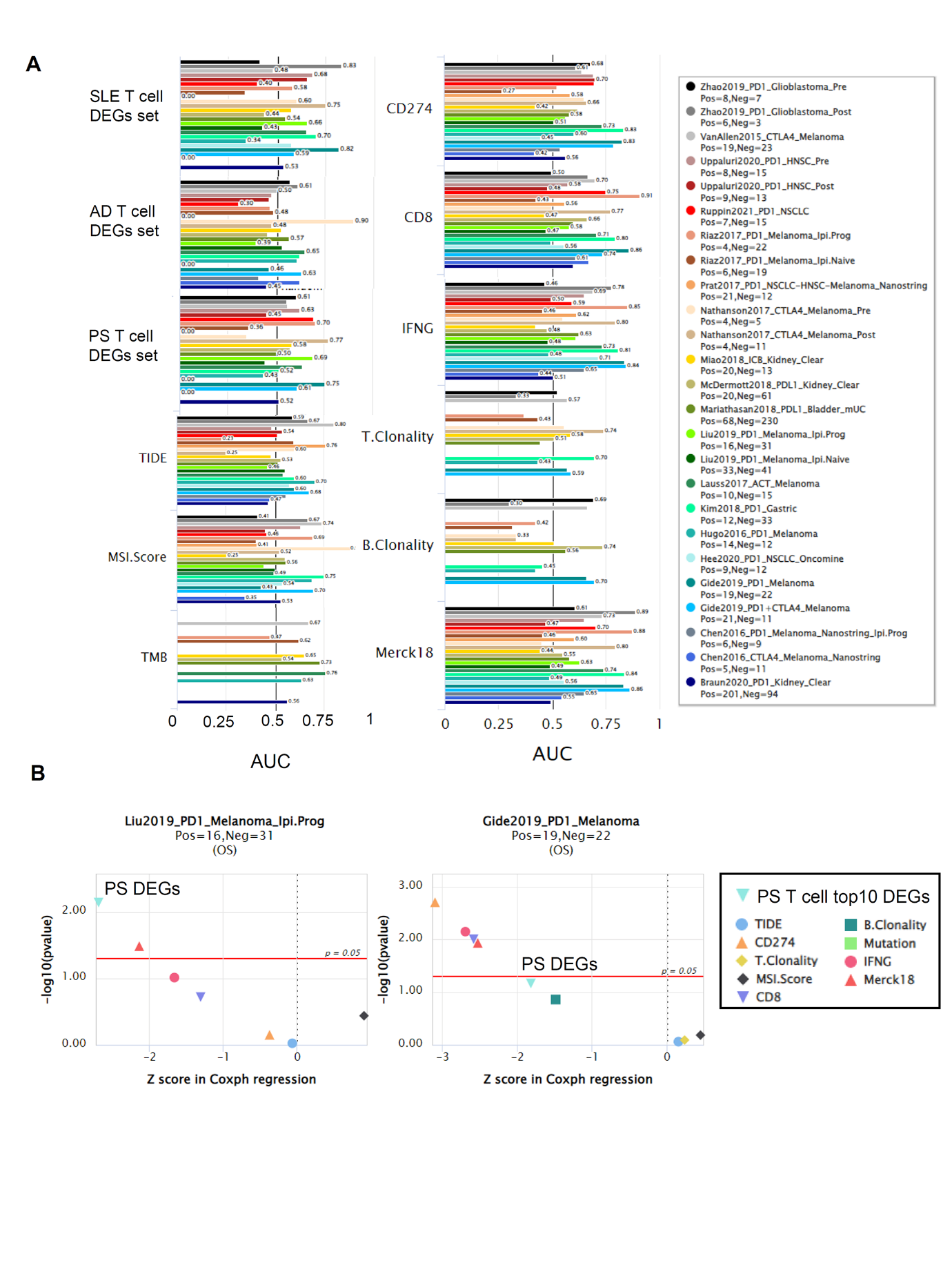
Supplementary Figure 6.

(A) Further analysis for the availability of SLE, AD, PS T cell DEGs set as biomarkers for prediction of ICB treatment prognosis in melanoma patients by TIDE; Wilcoxon rank sum test results of SLE, AD, PS T cell DEGs sets and other biomarkers in established melanoma patient data sets in TIDE, scored by Area Under the Curve (AUC).

(B) Statistical significance of PS T-cell DEG set in anti-PD-1 treated melanoma data sets.
